# Association between social jetlag and weight and fat reduction in dieting

**DOI:** 10.1007/s41105-024-00539-8

**Published:** 2024-06-24

**Authors:** Kai Minabe, Akiyoshi Shimura, Ko Sugiura, Hiroko Hino, Yusaku Akatsuka, Takeshi Seto, Miho Yanai, Jiro Masuya, Yu Tamada, Takeshi Inoue

**Affiliations:** 1 Department of Psychiatry, Tokyo Medical University, 6-7-1 Nishi-Shinjuku, Shinjuku-Ku, Tokyo 160-0023 Japan; 2 Department of Psychiatry and Behavioral Sciences, Stanford University, 3165 Porter Drive, Palo Alto, CA 94304 USA; 3 Department of Research and Development, Children & Future Co., Ltd, 6-16-4 Okusawa,, Setagaya-Ku, Tokyo 158-0083 Japan; 4 Department of Economics, University of Houston, Houston, TX 77004 USA; 5 RIZAP Inc, 8-17-1-36F Nishi-Shinjuku, Shinjuku-Ku, Tokyo 160-0023 Japan; 6 Department of Psychiatry, Tokyo Medical University Hachioji Medical Center, 1163 Tatemachi, Hachioji-Shi, Tokyo 193-0998 Japan

**Keywords:** Social jetlag, Chronobiology, Diet, Exercise, Nutrition, Obesity

## Abstract

Objectives: Social jetlag (SJL), the discrepancy between an individual’s inherent circadian rhythm and external social schedule, is associated with obesity. This study aimed to investigate whether SJL also influences body weight and body fat loss during dieting. Methods: This was an observational study from 2015 to 2018 with participants who had joined an exercise and nutrition program at a private personal training gym. Data from 11,829 individuals provided by the gym along with their sleep logs were analyzed. Differences in change in body mass index (BMI) and body fat percentage (%body fat) were compared by the degree of SJL. Regression was conducted for the change in BMI and %body fat on SJL, adjusted for gender, age, engagement duration in the program, initial BMI, initial %body fat, chronotype, and dietary intakes. Results: The subjects comprised 3,696 men and 8,133 women with a mean age of 40.4 years. Greater SJL was associated with a lower efficacy of BMI and %body fat reduction. The change in BMI (+ 0.56 / hour: SJL) and %body fat (+ 1.40 / hour: SJL) was associated with SJL after adjusting for each variable including dietary intake. Conclusion: SJL was associated with the effect of exercise and nutrition instruction on BMI and body fat reduction, even after adjustment for covariates related to dietary intake. Maintaining consistent sleep–wake rhythms may be crucial for enhancing the efficacy of weight loss programs.

**Supplementary Information:**

The online version contains supplementary material available at 10.1007/s41105-024-00539-8.

## Introduction

Obesity has rapidly increased globally over a period of 40 years since 1975, reaching a total of 640 million individuals (260 million men and 375 million women). The proportion of obese individuals has increased during this time to 11% in men (a threefold increase), and 15% in women (a twofold increase) [1]. It is well-known that obesity can lead to diabetes [2], hypertension and renal disease [3], cardiovascular disorders [4], gout [5], lipid abnormalities and fatty liver [6], obstructive sleep apnea syndrome [7], orthopedic disorders [8], menstrual irregularities [9] and pregnancy complications [10]. Additionally, it significantly increases the mortality rate [11]. Therefore, the management of obesity has become a public health issue.

Social jetlag (SJL) [12], refers to the mismatch between societal time and the body’s internal clock. Many individuals experience advances in wakeup times and sleep deprivation owing to societal constraints, such as work, school, and household responsibilities, and wake up using alarm clocks on “workdays”. On the other hand, on “freedays”, individuals experience recovery sleep, as they try to catch up on accumulated sleep deprivation by sleeping for prolonged periods. The mismatch in sleep timing between workdays and freedays leads to SJL. Previous studies [13, 14] have indicated a positive association between a high body-mass index (BMI, BMI of 25 or above is “overweight”) and SJL, suggesting that SJL may be a contributing factor to an individual’s present BMI. Furthermore, SJL has been shown to be associated with metabolic risk factors, such as low levels of high-density lipoprotein cholesterol and high levels of triglycerides and neutral fats [14, 15], as well as having a potential association with metabolic syndrome. In addition, SJL has been reported to potentially increase diurnal cortisol levels [16]. A 2-h SJL has been reported to increase the risk of prediabetes and type 2 diabetes by approximately twofold [17] and to be associated with impaired glucose homeostasis, increased fasting plasma insulin concentrations, and insulin resistance [15], thus affecting glucose tolerance. Other studies have noted a decrease in cardiac autonomic regulation as measured by heart rate variability in individuals with high levels of SJL [18], as well as potential associations with cardiovascular diseases, and depression [19]. Moreover, SJL has been reported to be associated with high levels of occupational stress, suggesting that it may be a social issue as well as a health issue [20].

There are limited data on the impact of SJL on efficacy of weight loss programs for addressing obesity. There is only one previous study to our knowledge that has investigated the influence of chronotype and sleep schedule on weight loss outcomes among participants in a weight loss program, which reported that the instability of sleep schedule (inter-daily stability) did not have an effect [21]. However, this previous study did not measure or assess SJL, so the effects of SJL on weight loss outcomes in individuals seeking to lose weight remain unclear. We therefore hypothesized that SJL, which is thought to have an effect on obesity and metabolism, influences the efficacy of weight loss when obese individuals attempt to lose weight. To test our hypothesis, we investigated the effects of SJL on weight and body fat loss in participants in a relatively large exercise and nutrition instruction program.

## Participants and methods

### Design

This was an observational study. The target sample size to be analyzed was set to around 20,000. As it was indicated that greater than 2 h of social jetlag resulted in poor health outcomes [22], previous studies revealed that the prevalence of people with SJL greater than 2 h is around 3.3–10% [23, 24]. To detect a weak effect (Cohen’s *d* = 0.2), 651 samples were required, meaning a parent population of around 6500–19500 is needed. Thus, this study was conducted once a sample size close to this target was collected.

## Subjects

The subjects of this study were 18,363 adults who participated in a weight loss program at a Japanese private personal training gym between 2015 and 2018. Participants were also instructed to record their sleep/wake times during the intervention. Of the 18,363 subjects, those who recorded their weight and body fat percentage only once or less were excluded from the analysis, and data from the remaining 11,829 subjects (64.4%) were analyzed. Upon joining the program and before starting their exercise and nutrition regimen, participants provided informed consent that their data might be used for future academic analyses in an anonymous manner. This study was conducted with approval from the Tokyo Medical University Medical Ethics Review Committee (study approval no.: T2018-0041), and anonymous data was provided by the private personal training gym company (RIZAP Inc., Tokyo, Japan).

## Diet program

The program targets weight and body fat reduction and comprises twice-weekly, 50-min resistance training sessions and individual face-to-face nutritional guidance. In the initial nutritional session, a dietary intake target is established. This daily caloric intake target is derived by subtracting 500–1000 kcal from the daily estimated calorie consumption, calculated based on the daily basal metabolic rate and physical activity level. Furthermore, the target daily caloric intake never falls below the basal metabolic rate. Participants are also educated about the significance of each nutritional component and the importance of maintaining a balanced intake. The personal training regimen emphasizes weight training and incorporates exercises primarily targeting major muscle groups. Participants engage in these 50-min workouts twice weekly at the gym.

## Measurements

Their profile data, including age and gender, and body measurements such as height, weight, estimated body fat mass, and percentage (%body fat) were registered in the data table. The participants recorded their sleep logs daily using the mobile application. Regarding dietary intake, the participants also input food items and quantities for each meal into the application, which are then reviewed by program instructors or nutritionists. The application estimates the nutrient intake levels for each food item, based on which the total nutritional intake for each meal is computed and recorded. Participants' body weight and estimated %body fat are measured during their visits to the exercise gym. The %body fat is determined using a bioelectrical impedance analysis machine, which has high validity and accuracy in estimating human body fat percentage, comparable to air displacement plethysmography [21]. There are no specific requirements or inclusion/exclusion criteria regarding the duration of participation in the program. The period from joining the program to the last data registration is defined as “engagement duration” in this study.

## Analysis

SJL, as defined by the Munich ChronoType Questionnaire (MCTQ) [25, 26], is calculated by subtracting the midpoint of sleep on free days (MSF) from the midpoint of sleep on workdays (MSW), and taking the absolute value of the resulting difference (SJL =| MSF – MSW |). Participants were divided into the following 3 groups based on the severity of their SJL: SJL < 1 h, 1 h ≤ SJL < 2 h, and 2 h ≤ SJL. One-way ANOVA followed by a post-hoc test (Tukey HSD) was used to compare the mean differences in SJL and reductions in BMI and %body fat between the groups. Additionally, multivariable regression analysis was performed to adjust for factors such as gender, age, engagement duration, initial BMI, initial %body fat, MSFsc: the indicator of chronotype, calculated from MSF with sleep-debt correction by the method of MCTQ, and dietary intake, to analyze reductions in BMI and %body fat. Statistical analyses were performed using IBM SPSS Statistics version 29 software. The statistical significance level was set at *p* < 0.05 and the 95% confidence interval, as mentioned in the design section.

## Sensitivity analyses

As sensitivity analyses, average sleep duration was also adjusted in sensitivity analyses. When adjusting for MSFsc, its value was converted to a floating-point number before being input into the regression analyses. Regarding the covariates in the multivariable model, gender was assessed on a categorical scale, while the others were assessed on a continuous scale.

## Results

### Demographics

Table 1 illustrates the characteristics of the study participants. The mean age was 40.4 (SD: ± 10.3) years, and 31.2% of the overall participants were men. The mean engagement duration in the program of the participants was 154.8 ± 168.5 days. The mean initial BMI was 27.0 ± 4.8 and the mean initial %body fat was 33.4% ± 8.1%. The overall mean SJL was 0.4 ± 0.5 h, with 89.2% of the participants in the group with SJL < 1 h, 9.3% in the group with 1 h ≤ SJL < 2 h, and 1.5% in the group with SJL ≥ 2 h. There were no significant differences in initial %body fat and height among the 3 groups. The correlations among variables were shown in Table 2.

**Table 1:** Demographic variables and differences in gender and social jetlag

	Total (*n* = 11,829)		Gender				SJL (Social jetlag)			
			Male (*n* = 3696)	Female (*n* = 8133)	*P *(T)		SJL < 1 h (*n* = 10,615)	1 h ≤ SJL < 2 h (*n* = 1053)	2 h ≤ SJL (*n* = 161)	*P *(F)
Age (years old)	40.4 (10.3)		42.3 (9.3)	39.6 (10.6)	***		40.8 (10.3)	37.6 (10.1)	36.5 (9.9)	***
Engagement duration (days)	154.8 (168.5)		149.2 (167.5)	157.3 (168.9)	*		159.5 (171.1)	115.3 (138.2)	104.2 (128.8)	***
Body measurement at the first session										
Body height (cm)	162.8 (8.2)		171.8 (6.1)	158.8 (5.4)	***		162.9 (8.3)	162.5 (8.2)	162.4 (8.2)	
Body weight (kg)	72.0 (15.8)		84.4 (15.0)	66.4 (12.6)	***		72.2 (15.8)	71.2 (15.3)	69.3 (13.3)	*
BMI (kg/m^2^)	27.0 (4.8)		28.6 (4.7)	26.3 (4.7)	***		27.1 (4.9)	26.8 (4.7)	26.2 (4.3)	*
%Body fat	33.4 (8.1)		27.1 (6.1)	36.3 (7.1)	***		33.4 (8.0)	33.6 (8.0)	32.2 (8.5)	
Body measurement changes after the observation period										
Δ Body weight / BMI (%)	− 8.6 (6.8)		− 9.0 (6.3)	− 8.4 (7.0)	***		− 8.8 (6.9)	− 7.4 (6.2)	− 5.9 (6.3)	***
Δ% Body fat	− 19.1 (16.4)		− 21.8 (17.7)	− 17.8 (15.6)	***		− 19.5 (16.5)	− 15.9 (15.0)	− 12.8 (18.1)	***
Daily food consumption during the program										
Total calorific intake (kcal)	1352.1 (209.8)		1473.1 (243.8)	1280.2 (144.9)	***		1352.4 (210.9)	1344.2 (194.8)	1383.7 (233.3)	
Carbohydrate intake (g)	53.3 (29.5)		54.9 (36.5)	52.4 (24.3)	*		53.5 (29.3)	51.2 (29.2)	55.0 (41.7)	
Fat intake (g)	81.3 (15.5)		88.6 (17.7)	77.0 (12.2)	***		81.2 (15.6)	82.2 (14.8)	84.2 (14.0)	
Protein intake (g)	101.7 (20.5)		114.0 (21.5)	94.4 (15.8)	***		101.9 (20.5)	99.9 (20.2)	101.6 (19.9)	
Sleep schedules										
Sleep onset on workdays	0:07 (1:19)		0:07 (1:20)	0:07 (1:19)			24:05 (1:15)	24:19 (1:32)	25:05 (2:35)	***
Wakeup on workdays	6:53 (1:13)		6:51 (1:13)	6:54 (1:13)	*		6:50 (1:09)	7:09 (1:25)	8:14 (2:14)	***
Sleep duration on workdays	6:39 (0:54)		6:37 (0:51)	6:39 (0:55)	*		6:38 (0:52)	6:41 (0:57)	7:00 (1:28)	***
Sleep onset on freedays	0:08 (1:24)		0:03 (1:25)	0:11 (1:23)	***		0:05 (1:19)	0:33 (1:40)	1:32 (2:53)	***
Wakeup on freedays	7:10 (1:17)		7:06 (1:18)	7:11 (1:17)	***		7:04 (1:11)	7:44 (1:33)	9:09 (2:37)	***
Sleep duration on freedays	6:54 (1:00)		6:56 (0:59)	6:53 (1:01)	*		6:53 (0:57)	7:03 (1:08)	7:29 (2:26)	***
Social jetlag	0:24 (0:33)		0:23 (0:35)	0:24 (0:31)			0:16 (0:15)	1:15 (0:17)	3:15 (1:45)	***
MSFsc	3:36 (1:12)		3:31 (1:14)	3:39 (1:11)	***		3:33 (1:08)	4:00 (1:25)	5:02 (2:15)	***

*BMI* Body Mass Index. *MSFsc* Midoint of Sleep on Free-days sleep-corrected. *p < 0.05, **p < 0.01, ***p < 0.001; there was a significantly difference among the group (*T*-test or one-way ANOVA)

**Table 2:** Correlation between observed variables

	**1**	**2**	3	4	5	6	7	8	9	10	11	12	13	14	15	16	17	18
1. ΔBody weight / BMI	–																	
2. Δ%Body fat	.791^**^	–																
3. Age	− .125^**^	− .142^**^	–															
4. Engagement duration (days)	− .189^**^	− .176^**^	.142^**^	–														
5. Initial BMI	− .378^**^	− .175^**^	− .016	.064^**^	–													
6. Initial Body fat	− .294^**^	− .115^**^	− .081^**^	.058^**^	.623^**^	–												
7. Total caloric intake	.071^**^	.059^**^	.073^**^	.036^*^	.136^**^	− .241^**^	–											
8. Carbohydrate intake	.103^**^	.064^**^	.163^**^	.205^**^	− .140^**^	− .159^**^	.542^**^	–										
9. Fat intake	.074^**^	.092^**^	− .030	− .085^**^	.173^**^	− .139^**^	.720^**^	− .052^**^	–									
10. Protein intake	− .091^**^	− .100^**^	.002	− .058^**^	.255^**^	− .153^**^	.555^**^	.038^*^	.215^**^	–								
11. Sleep onset on workdays	.045^**^	.046^**^	− .181^**^	− .009	.000	.002	.013	− .013	.045^**^	− .025	–							
12. Wakeup on workdays	.073^**^	.087^**^	− .295^**^	− .073^**^	.003	.042^**^	− .006	− .060^**^	.051^**^	− .014	.731^**^	–						
13. Sleep duration on workdays	.045^**^	.063^**^	− .161^**^	− .090^**^	.009	.049^**^	− .024	− .071^**^	.016	.013	− .374^**^	.349^**^	–					
14. Sleep onset on freedays	.057^**^	.064^**^	− .202^**^	− .018^*^	.001	.035^**^	− .003	− .016	.035^*^	− .043^*^	.834^**^	.660^**^	− .240^**^	–				
15. Wakeup on freedays	.069^**^	.090^**^	− .297^**^	− .077^**^	.009	.052^**^	− .021	− .057^**^	.037^*^	− .035^*^	.636^**^	.834^**^	.253^**^	.700^**^	–			
16. Sleep duration on freedays	.021^*^	.039^**^	− .121^**^	− .079^**^	.014	.015	− .024	− .057^**^	.004	.012	− .272^**^	.197^**^	.643^**^	− .410^**^	.354^**^	–		
17. Social jet Lag	.083^**^	.078^**^	− .117^**^	− .091^**^	− .026^**^	− .016	.003	− .022	.025	− .004	.104^**^	.163^**^	.057^**^	.172^**^	.256^**^	.083^**^	–	
18. MSFsc	.060^**^	.071^**^	− .259^**^	− .047^**^	− .002	.049^**^	− .018	− .036^*^	.033	− .052^**^	.765^**^	.806^**^	.039^**^	.830^**^	.831^**^	− .032^**^	.198^**^	–

^*^p < 0.05; **p < 0.01; Pearson’s correlation coefficient (*r*)

**Fig. 1:**
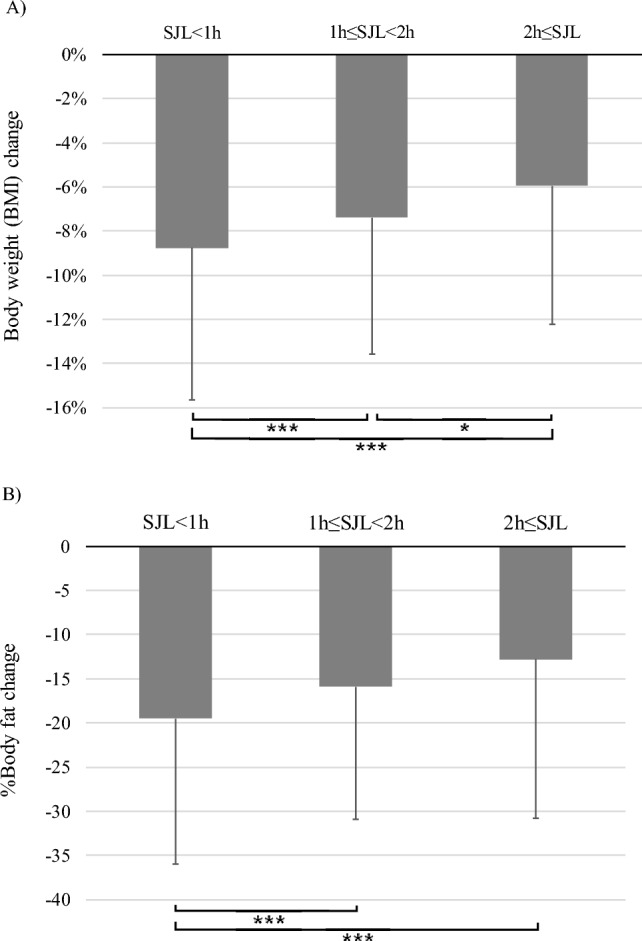
Body weight / fat change and Social Jet Lag. *p < 0.05, ***p < 0.001; ANOVA; post-hoc analysis (TUKEY-HSD)

Figure 1 shows a comparison of the decrease in BMI and %body fat among the 3 groups divided by SJL length (SJL < 1 h, 1 h ≤ SJL < 2 h, and 2 h ≤ SJL). Mean BMI reduction was 8.8% ± 6.9% (SJL < 1 h), 7.4% ± 6.2% (1 h ≤ SJL < 2 h), and 5.9% ± 6.3% (2 h ≤ SJL). Mean percent reduction in body fat was 19.5% ± 16.5% (SJL < 1 h), 15.9% ± 15.0% (1 h ≤ SJL < 2 h), and 12.8% ± 18.1% (2 h ≤ SJL). The results of one-way ANOVA and post-hoc analysis (Tukey HSD) demonstrated a significant trend towards a decreasing rate of BMI and body fat reduction with increasing SJL (*p* < 0.05).

## SJL and Diet

Table 3 shows the results of a multivariable regression analysis examining the effect of SJL on the BMI decrease rate, adjusting for gender, age, engagement duration, initial BMI, MSFsc, protein intake, carbohydrate intake, and fat intake. Model 1 adjusted for gender, age, engagement duration, and initial BMI. SJL had a significant effect on the change in BMI after adjustment (regression coefficient = 0.589%/hour, 95% Confidence interval (95% CI) = 0.212 to 0.966, *p* = 0.002). This model explained 17.4% of the change in BMI (adjusted *R*^2^ = 0.174). In Model 2, MSFsc was added as a covariate to the regression, and the results remained similar. SJL continued to affect the change in BMI (0.586%/hour, 95% CI = 0.204 to 0.968, *p* = 0.003, adjusted *R*^2^ = 0.174). In Model 3, protein intakes, carbohydrate intakes, and fat intakes were also adjusted additionally, and again, SJL continued to have an effect on the change in BMI (0.561%/hour, 95% CI = 0.189–0.933, *p* = 0.003, adjusted *R*^2^ = 0.196).

**Table 3:** Factors associated with body weight change

	Independent variables	Coefficients (%)	95% Confidence interval (%)	Standardized coefficients	*p*	VIF	Adjusted *R*^2^
Model 1	Gender (female vs. male)	− 0.845	− 1.315: − 0.374	− 0.056	< 0.001	1.083	0.174
	Age (/year)	− 0.064	− 0.088: − 0.041	− 0.085	< 0.001	1.077	
	Engagement duration (/day)	− 0.002	− 0.003: − 0.001	− 0.062	< 0.001	1.053	
	Initial BMI (/kg/m^2^)	− 0.601	− 0.647: − 0.555	− 0.403	< 0.001	1.051	
	Social jet Lag (/hour)	0.589	0.212: 0.966	0.048	0.002	1.017	
Model 2	Gender (female vs. male)	− 0.845	− 1.316: − 0.374	− 0.056	< 0.001	1.083	0.174
	Age (/year)	− 0.064	− 0.088: − 0.040	− 0.085	< 0.001	1.133	
	Engagement duration (/day)	− 0.002	− 0.003: − 0.001	− 0.062	< 0.001	1.053	
	Initial BMI (/kg/m^2^)	− 0.601	− 0.647: − 0.555	− 0.403	< 0.001	1.051	
	Social jet Lag (/hour)	0.586	0.204: 0.968	0.047	0.003	1.042	
	MSFsc (/hour)	0.009	− 0.181: 0.199	0.001	0.926	1.091	
Model 3	Gender (female vs. male)	− 0.262	− 0.804: 0.279	− 0.018	0.342	1.471	0.196
	Age (/year)	− 0.066	− 0.090: − 0.042	− 0.087	< 0.001	1.104	
	Engagement duration (/day)	− 0.002	− 0.003: − 0.001	− 0.073	< 0.001	1.099	
	Initial BMI (/kg/m^2^)	− 0.593	− 0.640: − 0.546	− 0.398	< 0.001	1.131	
	Social jet Lag (/hour)	0.561	0.189: 0.933	0.045	0.003	1.017	
	Carbohydrate intake (g/day)	0.021	0.013: 0.029	0.085	< 0.001	1.099	
	Fat intake (g/day)	0.064	0.049: 0.080	0.138	< 0.001	1.180	
	Protein intake (g/day)	− 0.012	− 0.024: 0.000	− 0.034	0.052	1.336	

*Model 2* Chronotype adjusted; *Model 3* Food consumption adjusted; *BMI* Body Mass Index; *MSFsc* Midoint of Sleep on free-days sleep-corrected

Table 4 shows the results of multivariable regression analysis to examine the effect of SJL on the decrease rate in body fat with adjusting gender, age, engagement duration, initial BMI, MSFsc, protein intakes, carbohydrate intakes, and fat intakes. Model 1 adjusted for gender, age, engagement duration, and initial %body fat. SJL had a significant effect on the change in %body fat after adjustment (regression coefficient = 1.371%/hour, 95% CI = 0.847 to 1.894, *p* < 0.001, adjusted *R*^2^ = 0.098) In Model 2, MSFsc was added as a covariate to the regression, and the results remained similar. SJL continued to affect the change in %body fat (1.214%/hour, 95% CI = 0.683 to 1.746, *p* < 0.001, adjusted *R*^2^ = 0.098). In Model 3, protein intakes, carbohydrate intakes, and fat intakes were also adjusted additionally, and again, SJL still had a significant effect on the change in %body fat (1.402%/hour, 95% CI = 0.456–2.348, *p* = 0.004, adjusted *R*^2^ = 0.114).

**Table 4:** Factors associated with body fat change

	Independent variables	Coefficients (%)	95% Confidence interval (%)	Standardized coefficients	*p*	VIF	Adjusted *R*^2^
Model 1	Gender (female vs. male)	8.095	7.377: 8.813	0.229	< 0.001	1.405	0.098
	Age (/year)	− 0.169	− 0.197: − 0.142	− 0.106	< 0.001	1.050	
	Engagement duration (/day)	− 0.014	− 0.016: − 0.013	− 0.148	< 0.001	1.031	
	Initial %body fat	− 0.480	− 0.521: − 0.438	− 0.236	< 0.001	1.398	
	Social jet Lag (/hour)	1.371	0.847: 1.894	0.045	< 0.001	1.021	
Model 2	Gender (female vs. male)	8.091	7.373: 8.808	0.229	< 0.001	1.405	0.098
	Age (/year)	− 0.158	− 0.187: − 0.129	− 0.099	< 0.001	1.112	
	Engagement duration (/day)	− 0.014	− 0.016: − 0.013	− 0.148	< 0.001	1.031	
	Initial %body fat	− 0.482	− 0.523: − 0.440	− 0.236	< 0.001	1.399	
	Social jet Lag (/hour)	1.214	0.683: 1.746	0.040	< 0.001	1.053	
	MSFsc (/hour)	0.412	0.166: 0.658	0.030	0.001	1.107	
Model 3	Gender (female vs. male)	10.363	8.734: 11.993	0.286	< 0.001	2.066	0.114
	Age (/year)	− 0.163	− 0.224: − 0.103	− 0.089	< 0.001	1.105	
	Engagement duration (/day)	− 0.006	− 0.008: − 0.003	− 0.078	< 0.001	1.096	
	Initial %body fat	− 0.592	− 0.676: − 0.508	− 0.274	< 0.001	1.529	
	Social jet Lag (/hour)	1.402	0.456: 2.348	0.047	0.004	1.017	
	Carbohydrate intake (g/day)	0.044	0.025: 0.064	0.075	< 0.001	1.105	
	Fat intake (g/day)	0.183	0.145: 0.221	0.162	< 0.001	1.174	
	Protein intake (g/day)	− 0.043	− 0.074: − 0.012	− 0.050	0.006	1.318	

*Model 2* Chronotype adjusted; *Model 3* Food consumption adjusted; *MSFsc* Midoint of Sleep on free-days sleep-corrected

## Sensitivity analysis

As sensitivity analysis, average sleep duration and chronotype (MSFsc) were adjusted (Supplemental Table 1 and Supplemental Table 2). With regard to sleep duration, it was not significant in body weight change and had tiny effects on body fat change. However, the effect disappeared after adjusting for food consumption, while SJL remained a significant factor. When adjusting for chronotype, both chronotype and SJL had a significant effect on body weight and body fat change, and SJL had a greater effect than chronotype.

## Discussion

In this study, we demonstrated that SJL was associated with a decreased effectiveness of exercise and nutritional instruction on weight and body fat loss. The effect of SJL on the decrease in body weight and fat was found to be significant even after considering the effects of gender, age, duration of observation, initial body weight and fat, chronotype, and dietary intake. SJL can be improved through increased awareness, and that it could be crucial in the pursuit of efficient dieting. Numerous previous studies have demonstrated an association between SJL and obesity; however, most of these studies were cross-sectional. Therefore, a longitudinal evaluation was necessary to clarify the association between SJL and obesity [27]. The present study, which analyzed outcomes after a certain period, offers a more definitive insight into the association than cross-sectional designs. Improving dietary habits and physical activity is crucial in addressing obesity. In this study, it was observed that a lower SJL is associated with the effects of these interventions, suggesting a potential public health implication.

Previous studies reported that individuals with a night-type chronotype are at a higher risk of obesity [28]. However, the mechanisms linking chronotype with obesity have not been clarified to date. In the present study, a multivariable analysis was performed with SJL and chronotype as explanatory variables, and MSFsc was not found to be a significant variable. Individuals with an eveningness chronotype are likely to have a greater SJL, which could act as a confounding factor. It is possible that the increased SJL associated with eveningness contributes to weight loss resistance, rather than the mere presence of an eveningness chronotype. On the other hand, being a night-type or having short sleep duration is reported to contribute to weight gain through increased food intake [29]. In this study, the diet program provided detailed guidance on food intake, and the lack of a significant link between chronotype and food intake might have reduced its impact on changes in body weight and body fat. Additionally, sleep duration, which was also not detected as a significant factor in the sensitivity analysis, despite many studies indicating that short sleep duration tends to lead to obesity [30, 31], may not have shown significance for similar reasons.

Compared with recent studies, the mean value of SJL in this study population is low. In China, it has been reported that 17% of participants had an SJL exceeding 1 h [23], and in Germany, within a work environment that includes shift work, an average SJL of 1.96 h [32] has been documented. In a recent survey of the general Japanese population, the average SJL was 0.91 h, with 40% of participants exceeding 1 h [24]. However, in this study, the average SJL was 24 min (0.6 h), and only 10.3% exceeded 1 h. The reason for this difference is unknown. These studies indicate that younger individuals tend to have larger SJLs, our study included a few younger participants, which might have influenced the lower observed value. Additionally, there could be a sampling bias, as individuals able to attend a personal training gym might not be engaged in occupations that require much overtime work, potentially excluding those with extreme SJL values. This study has some more limitations. First, the data used in this study was self-reported, not using actigraphy or any other objective methods, which may compromise its accuracy. SJL and chronotypes were evaluated by using self-administrated clock times, and the objective internal circadian rhythm indicators, such as the time of dim light melatonin onset, rectal temperature, or clock gene expressions, were not directly measured. Moreover, when using MTCQ methods to calculate SJL and MSFsc, the data from potential shift-workers were not excluded since the occupation and work styles of participants were not assessed in this study. Although the distribution of SJL was in a relatively lower and narrow range, the inaccuracies of these indicators still remain. Seconds, the adequacy of dietary restrictions and the heterogeneity of training loads among participants is unknown. The mean self-reported average daily total caloric intakes were low, suggesting high compliance with this weight loss program. However, the fidelity of the intervention was not directly assessed in this study and was not adjusted in the analyses. Furthermore, the baseline dietary intakes and physical activity levels before the intervention were also unknown. These data were not adjusted in this study. Third, there may be other confounders. The absolute value of SJL is likely to be larger among shift workers or participants with long working hours [33]. Those with limited free time may not have undergone sufficient physical activity in their daily life outside of the program’s training. Furthermore, not only SJL based on sleep–wake schedules, but also whole life schedule including mealtime schedules could affect dietary outcomes. Variable eating patterns cause adiposity and worsen glycemic control [34], which might contribute to obesity and adverse dietary outcomes. There may also be other lifestyle factors that increase SJL and contribute to the worsening of obesity, but not all potential confounding factors were investigated in this study. Fourth, this this study consists of a large sample size with over 10,000 participants, and not restricted to any specific clinical populations other than obesity, suggesting that its external validity may be relatively robust. However, the study was conducted among Japanese individuals, introducing an inherent racial bias. The extent to which these findings can be directly applied to other racial or ethnic groups remains uncertain, highlighting the need for future research in diverse populations.

Several limitations underlie in this study. First, a significant challenge is the unclear mechanisms of these results. Existing studies have suggested a correlation between SJL and increased insulin resistance and insulin secretion [15], as well as elevated cortisol levels [16]. However, this study did not investigate the physiological changes associated with increased SJL. Second, it is unclear whether these results can be generalized to all populations. This is because the study was conducted only in Japan, mainly composed of East Asian people, and did not examine the influence of ethnicity on the negative effects of SJL on obesity and metabolism. Third, although food consumption was adjusted for in the analyses, other factors that may affect diet, obesity, and health, such as alcohol intake, tobacco use, or any other supplement intake, were not examined. Especially in the case of alcohol intake, weekend drinking could be associated not only with obesity and dieting but also with the occurrence of social jet lag (SJL). Measuring and adjusting for these factors in future studies will be needed. Forth, this study is an observational survey and does not involve a randomized comparative trial. Therefore, it does not provide complete proof of a causal association between SJL and obesity. Specifically, a randomized controlled trial comparing a group in which SJL is reduced through guidance with a control group, while controlling for various conditions, is needed to determine whether improving SJL promotes improvement in obesity.

## Conclusions

Social jetlag, the misalignment between inherent circadian sleep–wake rhythms and socially required sleep schedules, was associated with the effect of exercise and nutrition instruction on BMI and body fat reduction, even after adjustment for covariates. Maintaining appropriate sleep–wake rhythms may play a role in the pursuit of efficient dieting.

## Supplementary Information

Below is the link to the electronic supplementary material.

Supplementary file1 (XLSX 17 KB)

## Declarations

### Research involving

Human Participants.
